# Trunk Muscle Coactivation in People with and without Low Back Pain during Fatiguing Frequency-Dependent Lifting Activities

**DOI:** 10.3390/s22041417

**Published:** 2022-02-12

**Authors:** Tiwana Varrecchia, Silvia Conforto, Alessandro Marco De Nunzio, Francesco Draicchio, Deborah Falla, Alberto Ranavolo

**Affiliations:** 1 Department of Occupational and Environmental Medicine, Epidemiology and Hygiene, INAIL, 00078 Rome, Italy; t.varrecchia@inail.it (T.V.); f.draicchio@inail.it (F.D.); a.ranavolo@inail.it (A.R.); 2 Department of Industrial, Electronic and Mechanical Engineering, Roma Tre University, 00146 Rome, Italy; 3 Department of Sport and Exercise Science, LUNEX International University of Health, Exercise and Sports, 4671 Luxembourg, Luxembourg; alessandro.denunzio@lunex-university.net; 4 Luxembourg Health & Sport Sciences Research Institute A.s.b.l., 4671 Luxembourg, Luxembourg; 5 Centre of Precision Rehabilitation for Spinal Pain (CPR Spine), School of Sport, Exercise and Rehabilitation Sciences, University of Birmingham, Birmingham B15 2TT, UK; D.Falla@bham.ac.uk

**Keywords:** fatiguing frequency-dependent lifting, low back pain, trunk muscle coactivation, sEMG

## Abstract

Lifting tasks are manual material-handling activities and are commonly associated with work-related low back disorders. Instrument-based assessment tools are used to quantitatively assess the biomechanical risk associated with lifting activities. This study aims at highlighting different motor strategies in people with and without low back pain (LBP) during fatiguing frequency-dependent lifting tasks by using parameters of muscle coactivation. A total of 15 healthy controls (HC) and eight people with LBP performed three lifting tasks with a progressively increasing lifting index (LI), each lasting 15 min. Bilaterally erector spinae longissimus (ESL) activity and rectus abdominis superior (RAS) were recorded using bipolar surface electromyography systems (sEMG), and the time-varying multi-muscle coactivation function (TMCf) was computed. The TMCf can significantly discriminate each pair of LI and it is higher in LBP than HC. Collectively, our findings suggest that it is possible to identify different motor strategies between people with and without LBP. The main finding shows that LBP, to counteract pain, coactivates the trunk muscles more than HC, thereby adopting a strategy that is stiffer and more fatiguing.

## 1. Introduction

Lifting tasks are manual material-handling activities and are commonly associated with work-related low back disorders (WLBDs) [1,2,3], which include both low back pain (LBP) and low back injuries. An accurate and precise biomechanical-risk assessment allows not only for the prevention of the onset of WLBDs but also an evaluation of the effectiveness of ergonomic interventions [3,4,5,6,7,8,9], i.e., redesign the working environment or work station [10] used to reduce WLBDs occurrences and costs [11]. 

In recent years, to integrate the Revised National Institute for Occupational Safety and Health (NIOSH) Lifting Equation (RNLE), which is the most widely used approach for the biomechanical risk assessment of lifting heavy loads [2,12,13,14], instrument-based tools have been designed and developed [15]. These quantitative approaches, which have been further optimized with machine-learning techniques [16,17], rely on kinematic, kinetic and surface electromyography (sEMG) indexes (i.e., such as mechanical energy consumption, compression and shear forces on the spine and trunk muscle coactivation) associated with different lifting risk conditions which are positively correlated to compressive and shear forces at the lumbosacral region of the spine [15,18,19,20]. 

The above-mentioned indexes have significant advantages, as they can be used in scenarios in which RNLE cannot, their calculation has a very low computational cost, and the sensors used to record the signals from the human body are unobtrusive, wireless, wearable, miniaturized, and have low power consumption [15]. However, they have never been tested in workers with LBP. When LBP occurs, many workers continue to work despite pain, exposing themselves to an unknown risk [21]. The presence of LBP commonly implies the adoption of different motor strategies (e.g., stiffening the spine, avoiding motion and increasing trunk reflex gains [22]) typically aimed at reducing the pain [23]. A common strategy adopted is to increase trunk stiffness, most likely due to augmented trunk muscle activity and changes in the reflex control of trunk muscles. This mechanism, which intends to protect the spinal structures, could have long-term consequences for spinal health and pain recurrence due to decreased damping compromising trunk dynamics [24]. Trunk stiffness is increased by increasing antagonist trunk-muscle coactivation [18,20,25,26], which is a common adaptation in people with LBP, seen in various conditions, even in standing [27]. Several approaches were proposed to estimate muscle coactivation [28], and they have been applied in different experimental studies. Studies have revealed that antagonist muscle activity counteracts the agonist actions producing functionally unfavorable moments that do not contribute to the required net trunk moment [29,30,31,32,33,34,35,36]. Furthermore, increased muscle coactivation generates increased compressive and shear forces across the spine [37,38,39,40] and an increased risk of WLBDs [25,41,42,43]. On the other hand, other studies have shown that during lifting tasks, the coactivation of the trunk muscles increases as the level of risk increases to improve spine stability and prevent the development of LBDs [15,20,25].

The biomechanical risk has been studied mainly in single frequency-independent lifting tasks with no adjustments for the influence of muscle fatigue. Just recently, a study carried out by our group provided the first risk assessment for fatiguing frequency-dependent lifting tasks [44] based on bipolar and high-density surface electromyography parameters. 

The current study aimed to highlight motor strategies by comparing trunk muscle coactivation in people with and without LBP during the execution of fatiguing frequency-dependent lifting tasks at three increasing levels of risks. The time-varying multi-muscle coactivation function (TMCf) [18] was selected as the method to compute muscle coactivation. We hypothesized that people with LBP will show a higher level of muscle coactivation than asymptomatic participants and will develop muscle fatigue at a faster rate.

## 2. Materials and Methods

### 2.1. Participants

Fifteen healthy control (HC) participants (nine females and six males; age: 27.87 ± 3.98 years; body mass index [BMI]: 25.26 ± 3.21 kg/m^2^) and eight (four females and four males; age: 25.15 ± 6.5 years; BMI: 23.51 ± 4.59 kg/m^2^) people with LBP were enrolled. All of the participants with LBP reported pain in the low lumbar region. Research brochures were distributed and people contacted us if they were interested in taking part in the study. Before enrolling, we confirmed that the study’s inclusion requirements were met using a standardized questionnaire. The following eligibility criteria were used:

- capacity to give informed written consent;
- no concurrent systemic, rheumatic or neuro-musculoskeletal disorders, which may confound testing, or on high doses of opioids (>30 mg of morphine equivalent dose);
- no current pregnancy;
- HC did not have a relevant history, over the last three years, of back and lower-limb pain or injury that limited their daily activities and/or required treatment from a health professional;
- LBP participants presented with chronic pain for at least 3 months during the past 6 months, which was not attributed to a specific pathology.
- LBP participants had not received treatment from a therapist in the last three months before the date of enrolment.

Before taking part in the study, all participants provided written informed consent, which was carried out in accordance with the Declaration of Helsinki at the Centre of Precision Rehabilitation for Spinal Pain (CPR Spine), the University of Birmingham, United Kingdom, and approved by the School of Sport, Exercise & Rehabilitation Sciences Ethics Committee (protocol number MCR260319-1). To eliminate expectation bias, no information about the expected results was provided to the participants.

### 2.2. Experimental Procedure

**Figure 1:**
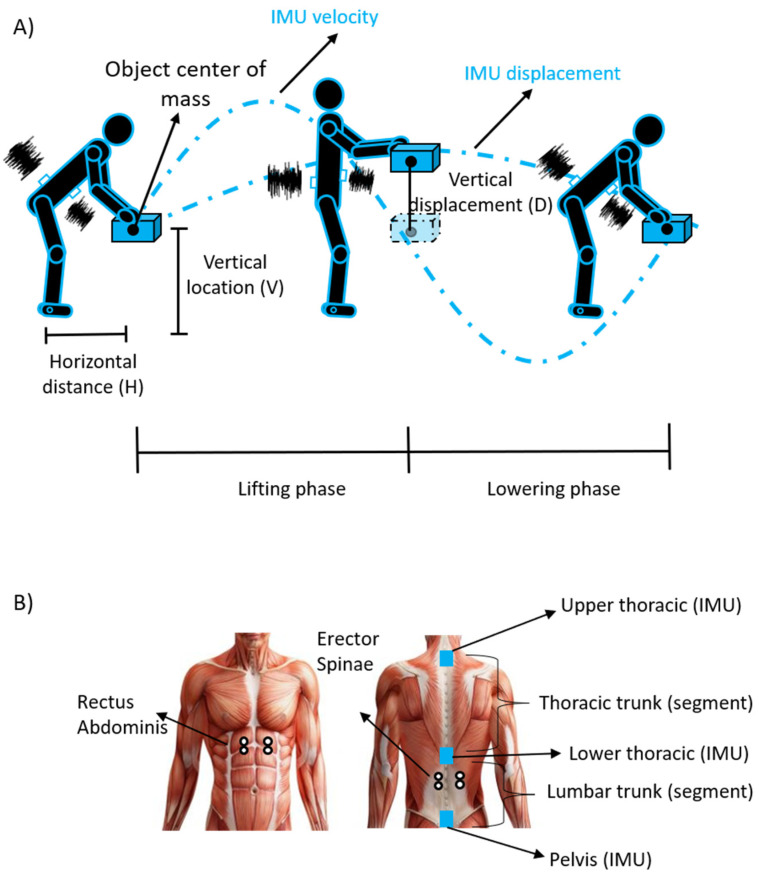
(**A**) description of the experimental setup and of the lifting cycle: displacement and velocity of the IMU placed on the load were used to define the lifting and lowering phases (see Section 2.5.1 for further details). (**B**) Locations of the IMU (blue squares) and sEMG (white circles) electrodes.

The experimental procedure presented in Varrecchia et al. 2021 was performed [44]. Briefly, the participants performed lifting tasks in three different lifting conditions (see Table 1) selected to obtain Lifting Index (LI) values of 1, 2, and 3 [12]. LI was calculated as follows:

(1) $$\mathrm{LI}=\frac{L}{\mathrm{RWL}}=\frac{L}{\mathrm{LC}\times \mathrm{HM}\times \mathrm{VM}\times \mathrm{DM}\times \mathrm{AM}\times \mathrm{FM}\times \mathrm{CM}}$$

where:

- L is the actual weight of the lifted load;
- RWL is the recommended weight limit that provides an estimate of the level of physical demand associated with the lifting task [12];
- LC is the constant load of 23 kg [12];
- HM, VM, DM and AM are the horizontal distance, vertical location, vertical displacement and asymmetry multipliers calculated by using equations or derived by tables by measuring the following parameters (see Figure 1A): horizontal distance (H); vertical location (V); vertical displacement (D); angle of asymmetry (A);
- CM is the coupling multiplier for the quality of gripping;
- FM is the frequency multiplier depending on lifting frequency (F), lifting duration and vertical location [12].

The three conditions differed only in the values attributed to F and FM in order to study the effect of frequency in this frequency-dependent task, while keeping the other NIOSH parameters constant for each risk condition (see Table 1). Notably, hand-to-object coupling was defined as “good” for all three lifting tasks [12].

Standing in a neutral body position [12,45] with the feet positioned parallel at a natural standing distance, the participants lifted the load (L = 10 kg, Table 1) represented by a plastic crate (34 × 29 × 13 cm) filled with a weight, using both hands in three distinct sessions, one for each LI, performed three different days. The different lifting sessions were randomized across the three sessions to avoid any confounding influence from a predefined order of the sequence of risk conditions. Each session was 72 h apart and was conducted at the same time of the day for each participant to avoid confounding effects due to fatigue or daily habits [46]. 

For a total lifting-task length of 15 min, the number of repetitions was determined by the frequency parameter utilized to obtain the specific LI for each session. Specifically, during the LI = 1, 2 and 3, 4, 11 and 15 lifts per minute were performed, respectively (Table 1).

Participants with LBP were asked to complete the lifting repetition until exhaustion if lasting less than 15 min. A timer and acoustic feedback were used to monitor the duration of lifting and the frequency of tasks, respectively. Specifically, each time the acoustic signal was heard, the participants raised the load to the defined height (V + D = 75 cm + 40 cm = 115 cm, Table 1), and then immediately lowered it again. Then, they released it by standing upright while waiting for the next acoustic signal. The task was performed with a self-selected strategy and no instructions were provided for the technique of task execution (e.g., bending of the trunk or limbs). In each of the three sessions, before the lifting tasks were performed, isometric maximum voluntary contractions (iMVCs) were performed for the trunk flexor and extensor muscles [47].

### 2.3. Electromyographic and Inertial Measurement Unit Recordings

Data from the bipolar sEMG and Inertial Measurement Unit (IMU) were acquired simultaneously. All devices were synchronized via a synching device (MyoSync, Noraxon, USA Inc., Scottsdale, AZ, USA).

#### 2.3.1. Bipolar sEMG

Four wireless bipolar sEMG sensors (Ultimium EMG system, Noraxon, USA Inc., Scottsdale, AZ, USA) were placed over the right and left erector spinae longissimus (RESL and LESL, see Figure 1B) and the right and left rectus abdominis superior (RRAS and LRAS, see Figure 1B) following the electrode placement guidelines [48] and the atlas of muscle innervation zones [49]. Before applying the sensors, the skin was prepared by shaving the area if needed, applying an abrasive paste (SPES Medica, Genova, Italy), and finally washing and drying the region. Then, the sensors were placed using bipolar disposable, wet-gel, self-adhesive Ag/AgCl snap electrodes (2 cm diameter; Dual EMG wet gel electrodes, Noraxon, USA Inc., Scottsdale, AZ, USA). The bipolar sEMG sampling frequency was set to 2000 Hz.

#### 2.3.2. Inertial Measurement Unit

Three inertial sensors (myoMotion Research PRO IMU, Noraxon) were used to acquire movements of the following body segments (Figure 1B): upper thoracic (T2), lower thoracic (over the spine at L1/T12) and pelvis (bony area of the sacrum at L5-S1 level). An additional IMU was placed on the plastic crate (z-axis in the vertical direction). Calibration was carried out with the participant in an upright standing position. The inertial sensor-sampling frequency was set at 2000 Hz, so as to be consistent with sEMG recordings. 

### 2.4. Questionnaires Data

At the end of each session, participants completed the Borg scale to rate fatigue (with 6–20 as anchor points for extremely light and extremely hard perceived exertion, respectively [50]). The pain level in the low back region was measured using a visual analogue scale (VAS, [51]) (with 0–100 as anchor points for no pain and the worst pain imaginable, respectively). In both groups, pain ratings were recorded before and after the session and every minute of the lifting exercise for individuals with LBP. 

### 2.5. Data Analysis

Data were processed using Matlab (version 2018b 9.5.0.1178774, MathWorks, Natick, MA, USA). The IMU and sEMG data during the lifting task were time-normalized to the duration of the lifting and lowering phases. A linear interpolation procedure was used to obtain 200 samples per phase to compare different lifting tasks with different durations [45].

#### 2.5.1. Lifting Cycles Detection

The vertical displacement and velocity of the IMU placed over the load were calculated by integrating the acceleration of the IMU (3rd order low-pass Butterworth filtered by applying a 10 Hz cut-off frequency, [44,52]) once and then twice, respectively, with the drift correction considering a null vertical acceleration and speed before and after the lifting action. Each whole-lifting cycle was subdivided into lifting and lowering phases. The onset and termination of the lifting phase were defined as the time point at which the IMU vertical velocity exceeded a threshold of 0.025 m/s and the peak of the IMU vertical displacement, respectively. The same threshold was used to set the termination of the lowering phase (see Figure 1A). After selecting the whole-lifting cycles, a Dynamic Time Warping approach [53] was used to align the curves that were shifted if wrong events were detected [44].

#### 2.5.2. Bipolar sEMG Preprocessing 

To decrease low-frequency artefacts and high-frequency noise, the sEMG signals recorded for both iMVC and tasks were band-pass filtered using a 3rd order Butterworth filter of 25–400 Hz [54,55]. Full-wave rectification and low-pass filtering using a 4th order Butterworth filter at 5 Hz were used to extract the envelope of sEMG signals of each lifting task [33]. The sEMG envelope was amplitude-normalized to the average iMVC peak value for each muscle [56,57,58,59].

#### 2.5.3. Time-Varying Multi-Muscle Coactivation Function (TMCf) 

The time-varying multi-muscle coactivation function (TMCf) [18,20] was computed to estimate the coactivation of the four trunk muscles during the lifting task using the following formula:

(2) $$TMCf\left(d\left(k\right),k\right)=\left(1-\frac{1}{1+e^{-12\left(d\left(k\right)-0.5\right)}}\right).\frac{\;(\sum_{m=1}^{M}sEMG_{m}\left(k\right)/M){\;}^{2}}{max_{m=1\ldots M}\left[sEMG_{m}\left(k\right)\right]}\;$$

where:

- d(k) is the mean of the differences between the *k*th samples of each pair of sEMG signals:

(3) $$d\left(k\right)=\frac{\sum_{m=1}^{M-1}\sum_{n=m+1}^{M}\left|sEMG_{m}\left(k\right)-sEMG_{n}\left(k\right)\right|}{J\left(M!/\left(2!\left(M-2\right)!\right)\right)}$$

- *J* is the length of the signal;
- *M* is the number of considered muscles;
- $sEMG_{m}\left(k\right)$ and $sEMG_{n}\left(k\right)$ are the *k*th sample value of the envelope of the sEMG signals of the m^th^ and nth muscles, respectively.

As coactivation indices, the mean (TMCf_Mean_) and the maximum (TMCf_Max_) values within the cycles were calculated. This function and indices were calculated within the lifting and lowering phase. 

TMCf_Mean_ and TMCf_Max_ in all the conditions (LI = 1, 2 and 3) of all of the lifting tasks were time-averaged across all the cycles and over one-minute consecutive windows to compare data with a different number of repetitions of the lifting cycles. The first five cycles of each lifting condition were averaged to compare the two groups in a non-fatigued condition. 

#### 2.5.4. Range of Motion and Trunk Stability Parameters

The flexion-extension range of motion (RoM) of thoracic (angle between upper thoracic and lower thoracic IMU) and lumbar (between lower thoracic and pelvis) trunk were extracted from the IMU system by calculating the difference between the maximum and minimum angle values within the lifting and lowering phase (RoM_Thoracic_ and RoM_Lumbar_). The stability parameters were extracted via the Root Mean Square (RMS) of the acceleration of upper (RMS_upper_) and lower (RMS_lower_) thoracic IMUs (see Figure 1B), an increase in which indicates a decrease in stability [52].

### 2.6. Statistical Analysis

The statistical analysis was performed using Matlab (version 2018b 9.5.0.1178774, MathWorks, Natick, MA, USA) to verify the difference between HC and LBP groups and the effect of the risk levels via the TMCf parameters across the total number of lifting repetitions, for the repetitions on the consecutive one-minute windows and separately for the lifting and lowering phases. The group effect was measured via the statistical analysis of the TMCf parameters for the first five cycles, as non-fatigued lifting cycles of each lifting condition. For each parameter, the normality of data distribution was checked using the Shapiro–Wilks test. Then, in each group (HC and LBP), one-way repeated-measures analysis of variance (ANOVA) or a corresponding Friedman t-test (if data not normally distributed) was performed to determine whether LI levels determine significant changes in each parameter. Post hoc analyses were performed using a paired t-test with Bonferroni’s corrections when significant differences were observed. For each LI, the unpaired two-sample t-test or Mann–Whitney (MW) test was used to evaluate differences in TMCf parameter between LBP and HC. The same statistical approach was used to verify the difference between HC and LBP groups, and the effect of the risk levels on and VAS and Borg scales. Statistical significance level was set as *p*-value < 0.05.

## 3. Results

### 3.1. TMCf 

**Figure 2:**
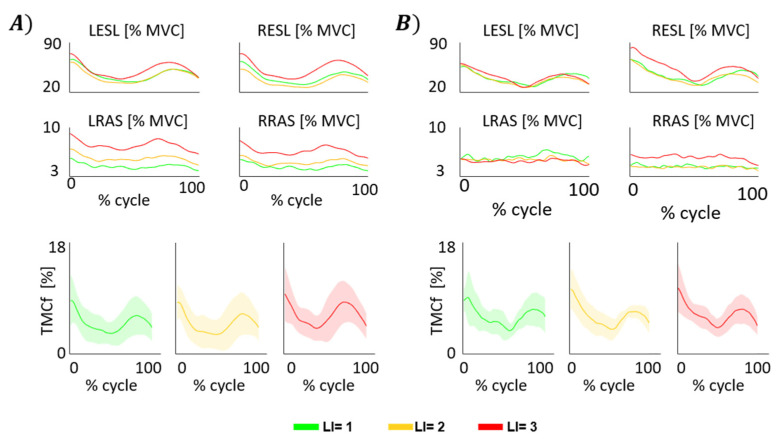
Mean envelopes among all subjects for each lifting condition of the left (LESL) and right (RESL) erector spinae longissimus and the left (LRAS) and right (RRAS) rectus abdominis, considering all the cycles of the task and mean envelopes (±SD) among all subjects for each lifting condition of TMCf function in both groups: healthy controls (**A**) and people with Low Back Pain (**B**). LI: Lifting index.

Figure 2 shows, for the HC (panel A) and LBP (panel B) groups, the mean envelopes of the LESL, RESL, LRAS and RRAS and the mean envelopes (±standard deviation, SD) of TMCf function among all subjects for each lifting condition, considering all the cycles of the task. 

**Figure 3:**
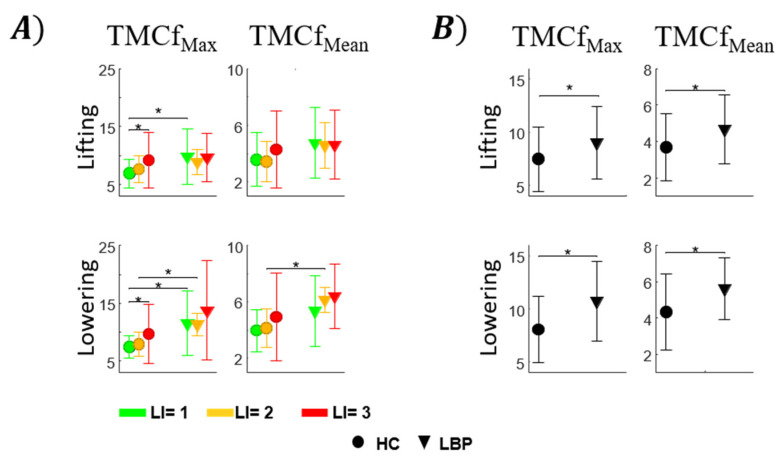
Mean ± SD for each risk level in both groups for the mean (TMCf_Mean_) and the maximum (TMCf_Max_) values of TMCf function considering all repetitions within the entire session, in lifting lowering phases (**A**) and considering the first 5 cycles for each lifting conditions (**B**). TMCf: Time-varying multi-muscle coactivation function. [* statistical significance (*p* < 0.05)].

Figure 3 shows the mean and standard deviation of TMCf_Max_ and TMCf_Mean_ for both groups considering the cycles in the total duration of the task for each lifting condition (panel A) and only the first five cycles of all conditions (panel B). For HC, statistically significant effects of LI for TMCf_Max_ in both lifting (F = 3.73, df = 2, *p* = 0.0367) and lowering (F = 3.49, df = 2, *p* = 0.044) phases were found while there was no significant effect for TMCf_Mean_ in both lifting (Chi = 2.13, df = 2, *p* = 0.344) and lowering (F = 1.9, df = 2, *p* = 0.169) phases (see Figure 3A). A post hoc analysis showed significant differences (*p* < 0.05) between LI = 1 and LI = 3 for TMCf_Max_ in both phases (Figure 3A).

For the LBP group, statistically significant effects of LI for TMCf_Max_ in both lifting (Chi = 0.25, df = 2, *p* = 0.883) and lowering (Chi = 0.75, df = 2, *p* = 0.687) as well as for TMCf_Mean_ in both lifting (F = 0.05, df = 2, *p* = 0.956) and lowering (F = 0.84, df = 2, *p* = 0.452) were found. Moreover, considering all the cycles in the total duration of the task (Figure 3A), statistically significant effects of group for TMCf_Max_ at LI = 1 in both lifting (*p* = 0.042) and lowering phase (*p* = 0.026), at LI = 2 in lowering phase (*p* < 0.001) and for TMCf_Mean_ at LI = 2 (*p* = 0.001) were found. Furthermore, considering only the first cycles of all conditions (Figure 3B), statistically significant effects of group for TMCf_Max_ in both lifting (*p* = 0.049) and lowering (*p* = 0.002) phases and for TMCf_Mean_ in both lifting (*p* = 0.036) and lowering phases (*p* = 0.012) were found.

**Figure 4:**
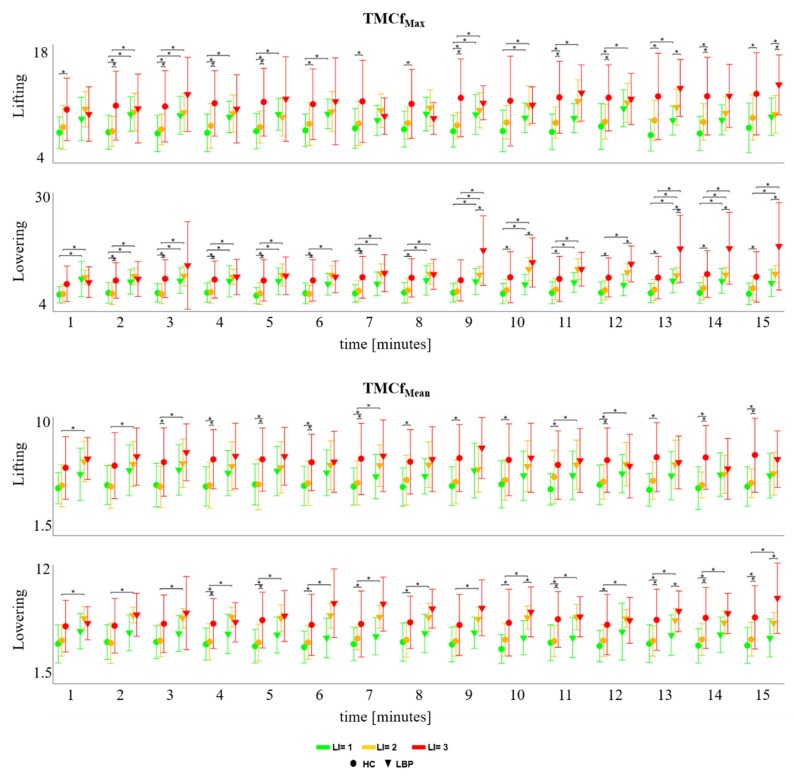
Mean ± SD for each risk level in both groups for the mean (TMCf_Mean_) and the maximum (TMCf_Max_) values of TMCf function considering all repetitions within each minute of lifting and lowering cycles. [* statistical significance (*p* < 0.05)].

Figure 4 shows the mean values and standard deviation of TMCf_Max_ and TMCf_Mean_ of all the participants for both groups and each LI during each minute of the task. For each period, statistically significant effects of LI (*p* < 0.05) for TMCf_Max_ and the TMCf_Mean_ were found. The statistical significances for the post hoc analysis are reported in Figure 4. Statistical differences were found between groups, as shown in Figure 4.

### 3.2. Trunk Motion 

**Figure 5:**
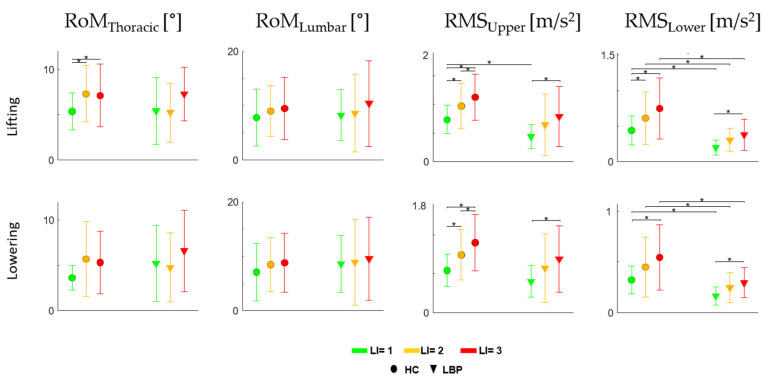
Mean ± SD for each risk level in both groups for the Range of Motion (RoM) of Thoracic (RoM_Thoracic_) and Lumbar (RoM_Lumbar_) regions and the Root mean square of the acceleration of upper (RMS_upper_) and lower trunk (RMS_lower_) values considering all repetitions within the entire session, in lifting and lowering phases. [* statistical significance (*p* < 0.05)].

Figure 5 shows the mean (±SD) of RoM_Thoracic_, RoM_Lumbar_, RMS_upper_ and RMS_lower_ for both groups, considering all the cycles in the total duration of the task for each lifting condition.

For the HC, the statistically significant effects of LI for RoM_Thoracic_ (F = 4.75, df = 2, *p* = 0.017) in the lifting phase, for RMS_upper_ in both lifting (F = 20.17, df = 2, *p* < 0.001) and lowering (F = 21.25, df = 2, *p* < 0.001) phases and for RMS_lower_ in both lifting (Chi = 17.73, df = 2, *p* < 0.001) and lowering phases (Chi = 12.13, df = 2, *p* = 0.002) were found.

For the LBP group, statistically significant effects of LI for RMS_upper_ in both lifting (Chi = 7, df = 2, *p* = 0.030) and lowering (Chi = 9.75, df = 2, *p* = 0.008) phases and for RMS_lower_ in both lifting (Chi = 7.75, df = 2, *p* = 0.021) and lowering (Chi = 9, df = 2, *p* = 0.011) phases were found. 

The post hoc analysis showed significant differences (*p* < 0.05) for HC between LI = 1 and LI = 2 and LI = 1 and LI = 3 for RoM_Thoracic_; for HC between each pair of LI (1 vs. 2, 2 vs. 3 and 1 vs. 3) for RMS_upper_ in both lifting and lowering phases; for HC between LI = 1 and LI = 3 for RMS_lower_ in both lifting and lowering phases and between LI = 1 and LI = 2 for RMS_lower_ in the lifting phase; for those with LBP between LI = 1 and LI = 3 for RMS_upper_ and RMS_lower_ in both the lifting and lowering phase (Figure 5).

Statistical significant differences between the groups (*p* < 0.05) for RMS_upper_ in the lifting phase at LI = 1 and for RMS_lower_ in lifting and lowering phases at LI = 1, LI = 2 and LI = 3 were found (Figure 5).

### 3.3. TMCf and Trunk Motion 

**Figure 6:**
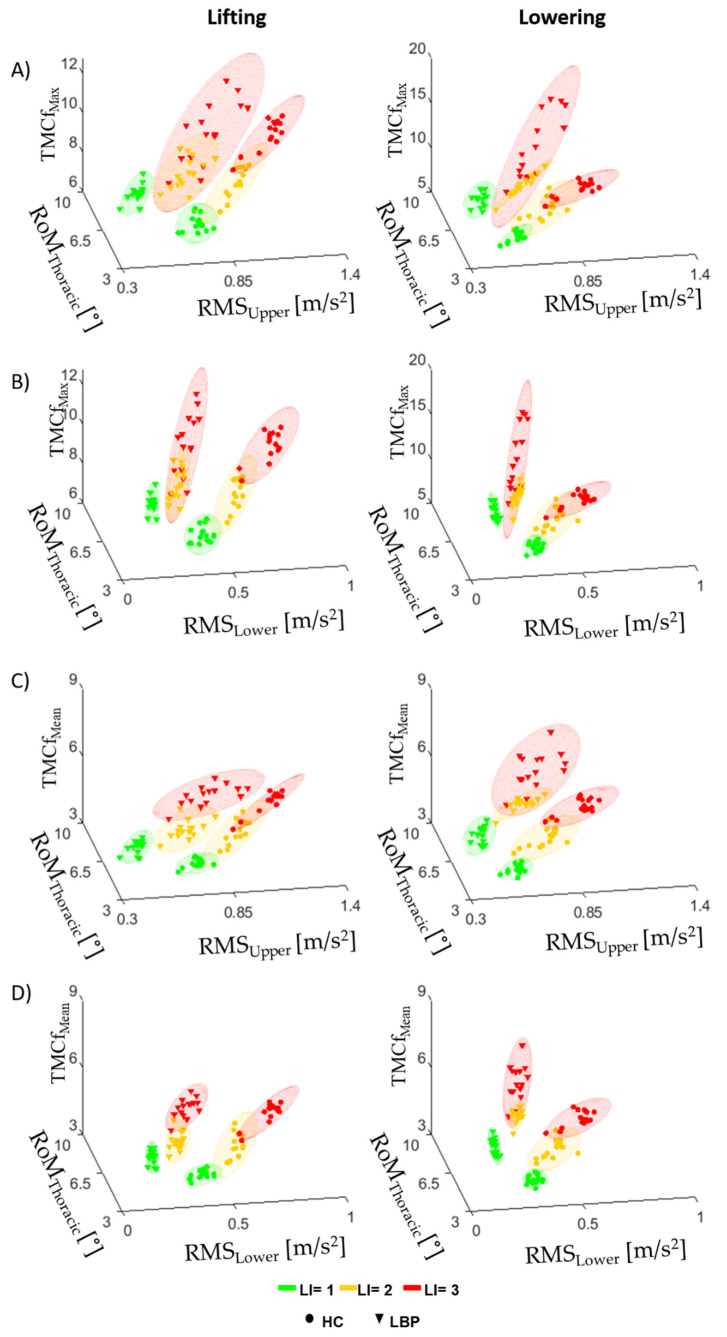
Plot 3D with mean for each risk level in both groups for the max (TMCf_Max_, (**A**,**B**) and mean (TMCf_Mean_, (**C**,**D**) values of TMCf function, the RoM of the flexion-extension of the Thoracic region (RoM_Thoracic_) and the RMS of the acceleration of the upper (RMS_upper_, (**A**,**C**) and lower trunk (RMS_lower_, (**B**,**D**) considering all repetitions within each minute of lifting and lowering cycles.

Figure 6 shows the mean values of TMCf_Max_ (Figure 6A,B) or TMCf_Mean_ (Figure 6C,D), with RoM of the trunk (RoM_Thoracic_) and the RMS of the trunk acceleration (RMS_upper_ in Figure 6A,C or RMS_lower_ in Figure 6B,D), considering all repetitions within each minute of lifting and lowering cycles for both groups.

### 3.4. Questionnaires

VAS and Borg scale average values at the end of each session are reported in Table 2 for both groups. For the HC, no significant effect of the LI was observed on perceived pain (*p* = 0.114), but there was a significant increase in perceived fatigue (*p* < 0.001). The post hoc analysis showed significant differences for perceived fatigue between LI = 1 and LI = 2 (*p* = 0.04) and between LI = 1 and LI = 3 (*p* = 0.002). For the LBP group, there was no significant effect of the LI on either pain intensity or perceived fatigue (*p* > 0.05). Statistically significant effects of LI = 1, LI = 2 (*p* < 0.01) and LI = 3 (*p* < 0.01) for pain intensity and of LI = 1 and LI = 3 (*p* = 0.04) for fatigue were found between the groups, while no significant effect of LI = 2 (*p* = 0.09) was observed for fatigue. 

**Figure 7:**
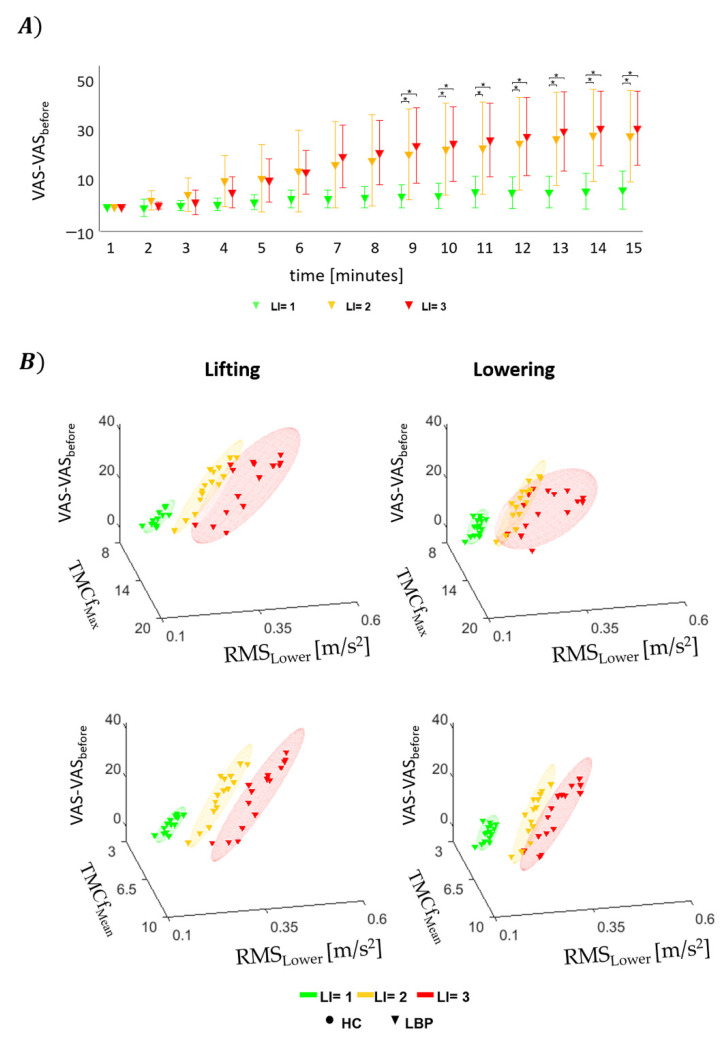
Mean values ± SD in each minute of VAS for pain intensity normalized to the values before starting the lifting (**A**). Plot 3D (**B**) with mean for each risk level in for the VAS, the max (TMCf_Max_) and mean (TMCf_Mean_) values of TMCf function and the RMS of the acceleration of lower trunk (RMS_lower_) considering all repetitions within each minute of lifting and lowering cycles. [* statistical significance (*p* < 0.05)].

Figure 7A shows the mean and standard deviation values in each minute for the VAS score for pain intensity normalized to the values before starting the lifting for those with LBP. Figure 7B shows the mean values of VAS values with TMCf_Max_ (first row) or TMCf_Mean_ (second row) and RMS_lower_ considering all repetitions within each minute of the lifting and lowering cycles for both groups. For each considered period, the statistical analysis revealed significant effects for VAS considering LI (*p* < 0.05). The statistical significances for the post hoc analysis are reported in Figure 7A.

## 4. Discussion

This study investigated trunk muscle coactivation and trunk movement strategies adopted by people with and without LBP during the execution of fatiguing frequency-dependent lifting tasks characterized by three different levels of risks. 

At the beginning of both the lifting and lowering phases, the TMCf showed high values with a reduction until the upright position was reached (end of lifting phase) and the load was released (end of lowering phase) (Figure 2). In addition, from a qualitative point of view, the curves representing the envelopes of LESL and RESL muscle activity and TMCf (Figure 2) are slightly different, and they show an earlier activation when the risk level increases.

When we analyse the first 5 cycles of the task, we notice that the coactivation of the trunk muscles of those with LBP is significantly higher than that of HC, independently from both the lifting index and phase of the task (lifting and lowering phases, Figure 3B). The increased values of both the mean and maximum of TMCf rely on increased muscle activity during the entire task duration. This can be interpreted as those with LBP, regardless of the fatiguing conditions, are likely exposed to more significant stresses at the L5-S1 segment (increased TMCf corresponds to an increased load at the L5-S1 joint [20]), and they undergo a greater risk for increased pain and injury [37,38,39,40,41,42,43].

Additional differences between people with and without LBP (Figure 3A) were found within each risk level (Figure 3A). Specifically, there was an increase in the maximum value of coactivation for those with LBP for LI = 1 in lifting and for LI = 1 and LI = 2 during the lowering phase. The results suggest that the lowering phase is more challenging for those with LBP, especially at low and medium risk levels, likely due to the eccentric nature of antagonist-muscle activation [60].

Both groups did not show statistically significant differences between the different levels of risk, except for differences between LI = 1 and LI = 3 for TMCf_Max_. This result reveals that the coactivation, calculated as an average across the entire task duration, does not vary across the risk levels as its time-varying nature is hidden by the averaging approach. On the contrary, the differences between the levels of risks for both groups and between groups were revealed by analysing the data on a minute-by-minute basis. These differences are particularly evident in the lowering phase, and they appear in both the maximum and the mean values (Figure 4), with the level of the maximum coactivation being constantly higher in those with LBP compared to the HC, especially during lowering. 

Considering trunk motion (RoM_Thoracic_ and RoM_Lumbar_ in Figure 5), the two groups have similar kinematic strategies and range of motion in each risk level (the only statistically significant difference was present for LI = 1 and both LI = 2 and 3 for HC during the lifting phase). This is widely justified by the geometry of the task, and was expected because the risk levels were different only for the lifting frequency. However, with the same kinematic strategy, the movement of those with LBP is much less efficient with a greater activation of the antagonist muscles [19], especially in the lowering phase. 

The evidence that the RRMS of IMU acceleration is significantly lower for those with LBP than for HC (Figure 5) can be justified by the consideration that the lower back, which is the main body segment involved during lifting and lowering tasks [61], is controlled by a strategy to minimize perturbations and so the reduction in this parameter implies an increase in stability [53]. 

A clustering approach (Figure 6) applied to the analyzed parameters (both muscular and kinematics) showed a significant difference between the two groups for each level of risk. Such a clustering approach will be applied and extended in future studies that aim to detect the onset of LBP based on the analysis of the trunk muscle and kinematic strategies adopted to reduce the biomechanical effort in lifting tasks. 

Differences in pain intensity (VAS) [51] and fatigue [50] between groups are consistent with the indexes of movement and muscular activity and allow different clustering results between the two groups.

The limitations of this study are the small sample size and the case–control study design. Future studies could consider larger sample sizes, other age groups, evaluate men and women separately and could also test other lifting conditions with the same LI values but with different multiplier values.

Collectively, our findings suggest that it is possible to identify different muscular and kinematics strategies between people with and without LBP: the main result shows that people with LBP coactivate their trunk muscles more than HC, by adopting a fatiguing trunk-stiffening strategy, possibly to avoid/minimize pain. This strategy implies an increased risk level that can be quantitively assessed using the trunk muscles coactivation indexes. 

The findings of this study provide a preliminary basis for future studies aiming to detect the onset of LBP via the TMCf assessment using a time-varying approach. 

## Figures and Tables

**Table 1:** For each task (A, B, and C), the values of the load constant (LC), the load weight (L), the horizontal (H) and vertical (V) locations, the vertical travel distance (D), the asymmetry angle (A), the lifting frequency (F) and the hand-to-object coupling (C) and the corresponding values of the multipliers (HM, VM, DM, AM, FM, CM), the recommended weight limit (RWL) and the lifting index (LI).

Task	LC (Kg)	H (Cm)	HM	V (Cm)	VM	D (Cm)	DM	A (°)	AM	F (Lift/Min)	FM	C	CM	L (KG)	RWL	LI
A	23	44	0.57	75	0.99	40	0.93	0	1	4	0.83	good	1	10	10	1
B	23	44	0.57	75	0.99	40	0.93	0	1	11	0.41	good	1	10	5	2
C	23	44	0.57	75	0.99	40	0.93	0	1	15	0.28	good	1	10	3.33	3

**Table 2:** Pain and fatigue scores measured at the end of each lifting task. VAS, visual analogue scale (0–100); HC: healthy controls; LBP: Low Back Pain participants; LI, Lifting Index. Values are presented as mean ± SD.

Scale	LI	HC (Mean ± SD)	LBP (Mean ± SD)
VAS Pain Intensity (0–100)	1	1.4 ± 3.22	42.25 ± 28.48
	2	4.73 ± 10.81	45.71 ± 17.7
	3	11.6 ± 22.78	45.4 ± 17.02
Borg Scale (6–20)	1	7.53 ± 1.55	10.13 ± 2.47
	2	9.2 ± 2.62	13.13 ± 1.96
	3	10.1 ± 2.65	13.5 ± 2.78

## Data Availability

The data presented in this study are available on request from the corresponding author.
